# Ebola virus infection induces autoimmunity against dsDNA and HSP60

**DOI:** 10.1038/srep42147

**Published:** 2017-02-09

**Authors:** H. Fausther-Bovendo, X. Qiu, S. McCorrister, G. Westmacott, P. Sandstrom, C. Castilletti, A. Di Caro, G. Ippolito, G. P. Kobinger

**Affiliations:** 1 University of Manitoba, Winnipeg, Canada; 2 National Microbiology Laboratory, Public health Agency of Canada, Winnipeg, Canada; 3 JC Wilt Infectious Disease Research Centre, Winnipeg, Canada; 4 National HIV and Retrovirology Laboratory, Ottawa, Canada; 5 Lazzaro Spallanzani, National Institute for Infectious Diseases-IRCCS, Rome, Italy; 6 Department of Pathology and Laboratory Medicine, University of Pennsylvania School 27 of Medicine, Philadelphia, PA, USA; 7 Laval University, Department of Microbiology and Immunology, Faculty of Medicine, Quebec, Canada.

## Abstract

Ebola virus (EBOV) survivors are affected by a variety of serious illnesses of unknown origin for years after viral clearance from the circulation. Identifying the causes of these persistent illnesses is paramount to develop appropriate therapeutic protocols. In this study, using mouse and non-human primates which survived EBOV challenge, ELISA, western blot, mass spectrometry and flow cytometry were used to screen for autoantibodies, identify their main targets, investigate the mechanism behind their induction and monitor autoantibodies accumulation in various tissues. In infected mice and NHP, polyclonal B cell activation and autoantigens secretion induced autoantibodies against dsDNA and heat shock protein 60 as well as antibody accumulation in tissues associated with long-term clinical manifestations in humans. Finally, the presence of these autoantibodies was confirmed in human EBOV survivors. Overall, this study supports the concept that autoimmunity is a causative parameter that contributes to the various illnesses observed in EBOV survivors.

The 2013–2016 Ebola virus (EBOV) outbreak has been the most devastating single outbreak on records claiming the lives of more than 11,000 individuals. Though the outbreak is now over, tremendous amount of work is still required to rebuild the medical infrastructures of the affected countries and to take care of the more than 17,000 survivors^1^,^2^. In addition to the stigma associated with survival, monitoring of EBOV survivors has shown that they suffer from various conditions months, even years after they are no longer viremic. Indeed, muscle and joint pains, as well as ocular diseases, hearing loss and mental health challenges (memory loss, confusion, sleeping disorders) were reported in EBOV survivors at higher frequency than the general population^3^,^4^,^5^,^6^,^7^. Some symptoms persisted for more than a year in more than a third of survivors^6^. It is worth noting that these long term sequelae denoted post EBOV disease syndrome (PEVDS) are observed in survivors after infection with various *ebolavirus* species including the highly lethal Zaire *ebolavirus*^3^,^4^,^5^,^7^ and the less lethal Bundibugyo *ebolavirus*^6^.

Unfortunately, the causes of these PEVDS remain unknown. EBOV has been detected in the central nervous system (cerebrospinal fluid), semen and eyes of some EBOV survivors up to 6, 13 and 14 weeks respectively after infection^5^,^7^,^8^. However, EBOV has not been detected outside of these immune privileged sites and no evidence suggests that these lingering viruses are responsible for PEVDS. The contribution of autoimmunity in PEVDS has not been investigated. Joint and muscle pains are common features of several autoimmune diseases such as rheumatoid arthritis and systemic lupus erythematosus^9^,^10^. Autoantibody production has been linked to a variety of ocular diseases^11^,^12^. Furthermore, successful treatments of ocular disease in EBOV survivors all involved atropine and corticosteroids administration^4^,^7^. The immunosuppressive properties of corticosteroids suggest that autoimmunity could be at least partially responsible for EBOV-induced ocular disease.

Here, we investigated the presence of autoantibodies in EBOV survivors using both the rodent and non-human primate (NHP) model of EBOV infection, as well as EBOV human survivors.

## Results

### Autoimmunity in mouse and NHP model of EBOV infection

**Figure 1:**
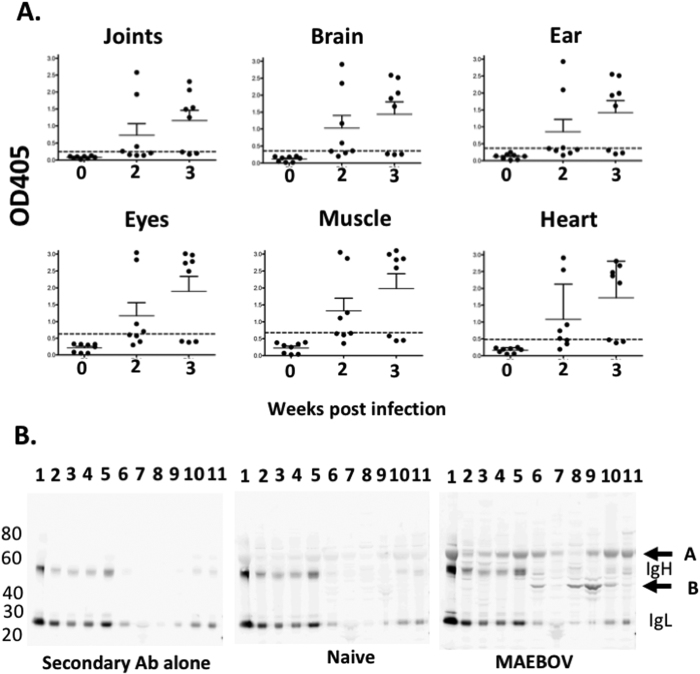
Autoantibodies are detected in some MA-EBOV surviving mice. Sera from naïve mice (**0**) and MA-EBOV infected mice 2 or 3 weeks after challenge were screened for the presence of autoantibodies against tissue lysate by ELISA (**A**) or WB (**B**). (**A**) For ELISA, sera were diluted 50 fold, cumulative results, from 2 separate experiments are depicted (n = 8 per group). Dotted lines indicate 3 times the mean OD value for naïve sera (**B**) For WB, the following tissue lysate were analyzed by WB; lane 1-blood, 2-spleen, 3-liver, 4-kidney, 5-lung, 6-joints, 7-eyes, 8-brain, 9-muscle 10-ear, 11-heart. Membranes were incubated with 500 fold diluted naïve or MA-EBOV survivor serum or secondary antibody alone (2ary) as control. Representative blots from 4 separate experiments.

First, the presence of autoantibodies in mice surviving mouse adapted (MA)-EBOV challenge was investigated. Forty mice were infected with a partially lethal dose (1LD50) of MA-EBOV and surviving mice were culled 2 or 3 weeks post challenge. Autoantibody levels were measured by ELISA using wells coated with lysates from various tissues from naïve mice. Autoantibodies against the tested tissues were detected in 25–50% and 62.5% of survivors 2 and 3 weeks post MA-EBOV challenge respectively, but not in control mice (Figs 1A and S1). Next, to determine whether one or several proteins in the tested tissues were targeted by these autoantibodies, binding of sera from naïve and MA-EBOV surviving mice to tissues lysate was analyzed by western blot (WB). Sera from naive mice did not strongly recognize any self-protein. Conversely, sera from MA-EBOV surviving mice predominantly recognized two targets, one around 60 kDa (target A) and another above 40 kDa (target B). Of note, the heavy (Ig_H_) and light chain (Ig_L_) of immunoglobulin (Ig) were also detected in several tissues by the goat anti-mouse IgG used as secondary antibody (Fig. 1B). The above results indicate that MA-EBOV survivors are prone to develop autoantibodies against various autoantigens.

In order to identify the 40 and 60 kDa autoantigens, joints, brain, muscle and ear lysates from naïve mice were separated by SDS-PAGE and stained using Coomasie blue. Bands of the appropriate molecular weights were excised from the SDS gels and analyzed by tandem mass spectrometry. Based on molecular weight and tissue distribution, heat shock protein (HSP) 60 was selected as candidate for the 60 kDa autoantigen while creatine kinase (CK) M and B type were selected for the 40 kDa autoantigen (Table S1).

**Figure 2:**
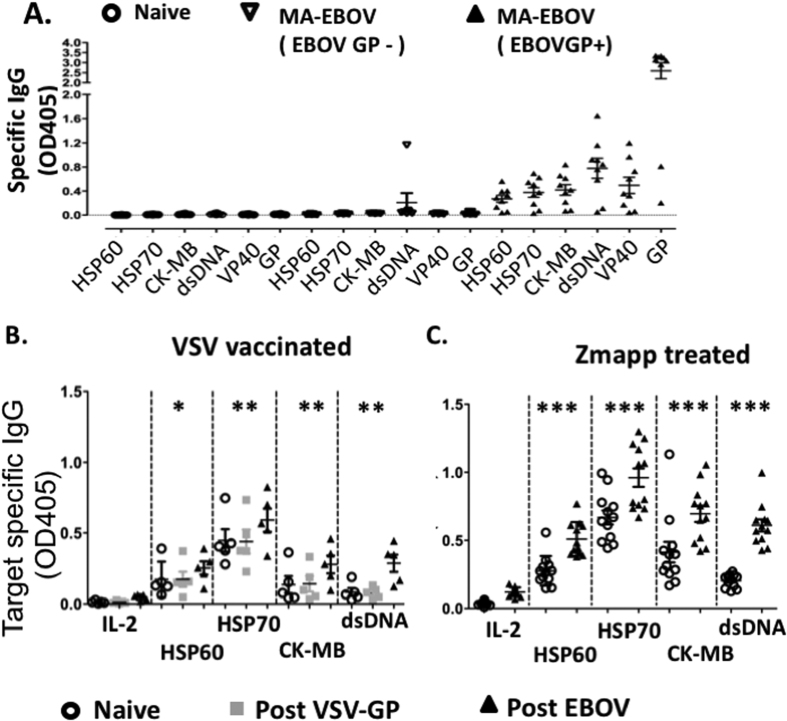
Polyclonal Autoimmunity is induced in rodent and NHP model of EBOV infection. Antibody levels against purified antigens were analyzed by ELISA in 200 fold diluted sera from mice (**A**) and NHP (**B,C**). Autoantibodies level were analyzed in naïve (n = 8) or MA-EBOV challenged mice (2 or 3 weeks post inoculation) (n = 16). Challenged mice were separated based on humoral response against GP. (**A**) Autoantibody levels were also monitored in NHPs (n = 5) before (open circles), 21 days post VSV-GP immunization (grey squares) and 26 days post EBOV challenge (filled triangles) (**B**). Autoantibody levels were in NHP before (open circle) and 28 days post EBOV challenge (filled triangles) in NHPs treated with ZMapp starting 4 or 5 days after challenge are also illustrated(n = 12) (**C**). Samples were analyzed in triplicate.

To confirm, the presence of autoantibodies against these candidates, wells were coated with purified proteins and the presence of antibodies against HSP 60 and CK MB isoform (CK-MB) was measured by ELISA. Furthermore, binding to HSP70 was also tested due to the role of anti-HSP70 antibodies in various autoimmune diseases^13^,^14^. EBOV glycoprotein (GP) and VP40 were used as positive controls. Not all MA-EBOV challenged mice developed antibodies against EBOV GP, suggesting that some mice were not productively infected following viral challenge. Inoculated mice were therefore separated based on the presence or absence of antibodies against EBOV GP. Autoantibodies against all the tested proteins were undetectable in naïve mice as well as MA-EBOV challenged mice which failed to develop a humoral response against EBOV GP, but detected in 78% (7/9) of challenged mice with detectable GP specific antibodies. Autoantibody levels against HSP70 and CK-MB were the highest, at level similar to anti-VP40 antibodies, but an order of magnitude lower than anti-GP antibodies level (Fig. 2A). It is worth noting that screening for autoantibodies by WB would not detect autoantibodies against double stranded (ds) DNA. Due to the crucial role of autoantibodies against dsDNA in systemic lupus erythematosus (SLE) an autoimmune disease^15^, level of autoantibodies against dsDNA were also measured in MA-EBOV survivors. 78% of surviving mice also developed high level of autoantibodies against dsDNA (Fig. 2A). These results suggest that a great proportion of MA-EBOV survivors develop autoantibodies against dsDNA, HSPs and CK-MB.

The NHP model of EBOV infection is considered the gold standard, as it more completely recapitulates the pathogenesis observed in humans. Therefore, the induction of autoantibodies was also evaluated in NHP infected with EBOV. Historical samples from ~1000LD50 EBOV challenged NHP which were either immunized with VSV encoding GP or treated with Zmapp were used^16^,^17^. Level of autoantibodies against HSPs, CK-MB and dsDNA were measured by ELISA before and 4 weeks post EBOV challenge. For VSV immunized NHP, autoantibody levels were also measured 21 days post vaccination (1 week before challenge). Autoantibody levels against IL-2 were used as negative control.

In VSV-GP immunized NHP, autoantibody levels against IL-2 were low or undetectable before, after vaccination or after EBOV challenge. Conversely, there was a statistically significant increase in autoantibody level against HSP60, HSP70, CK-MB or dsDNA, which was only detected after EBOV challenge but not post vaccination (Fig. 2B). Similarly, in Zmapp treated NHP, there was a statistically significant increase in autoantibody level against all tested autoantigens, with p values below 0.001 (Fig. 2B). It is also worth noting that the increase in autoantibodies level was likely not due to Zmapp, as the antibodies within this cocktail did not recognize any of the tested autoantigens (Fig. S2). In addition, autoantibodies induction could not be compared between VSV immunized and ZMapp treated NHPs as in naïve NHPs (prior to immunization or challenge), autoantibody background levels were lower in the VSV immunized group compared with the ZMapp treated one.

Overall, these experiments show that EBOV challenge induces autoantibodies against HSP, CK and dsDNA in survivors in both mice and NHP model of infection.

### Mechanism of EBOV-induced autoantibodies

To determine whether, autoantibodies against HSP, CK-MB and dsDNA were due to antigen mimicry, correlation between autoantibodies level and anti-EBOV GP was monitored. In mice, the level of antibodies against HSP60, HSP70, CK-MB and dsDNA strongly correlated with GP specific antibodies response (R^2^ values of 0.64, 0.67, 0.67 and 0.8 respectively and p values below 0.0001) (Figure S3). In NHP, an increase in autoantibodies against HSP60, HSP70 and CK were associated with stronger humoral responses against EBOV GP. However, a statistically significant correlation was only obtained between anti-EBOV GP titer and increase in HSP70 specific autoantibodies (p = 0.002, R^2^ = 0.64) (Figure S3).

Competition ELISA was then performed to demonstrate antigen mimicry between EBOV proteins and the tested autoantigens. Sera from MA-EBOV survivors with detectable autoantibodies were pre-incubated in presence or absence of gamma irradiated EBOV or Marburg virus (MARV). An hour later, sera were incubated with autoantigens coated plates. Interestingly, pre-incubating sera from MA-EBOV survivors with gamma irradiated EBOV, increased binding to CK, HSP60, HSP70 and dsDNA (Fig. S4), while decreasing binding to EBOV GP and VP40 (Fig. S4). It is worth noting that higher serum dilution was needed in order to observed a significant decrease in EBOV GP and VP40 binding, probably due to the higher level of these antibodies.

Although autoantibody levels against CK, HSP60, HSP70 and dsDNA correlate with GP specific response, these results indicate that they were not caused by antigen mimicry between EBOV proteins and the tested autoantigens.

**Figure 3:**
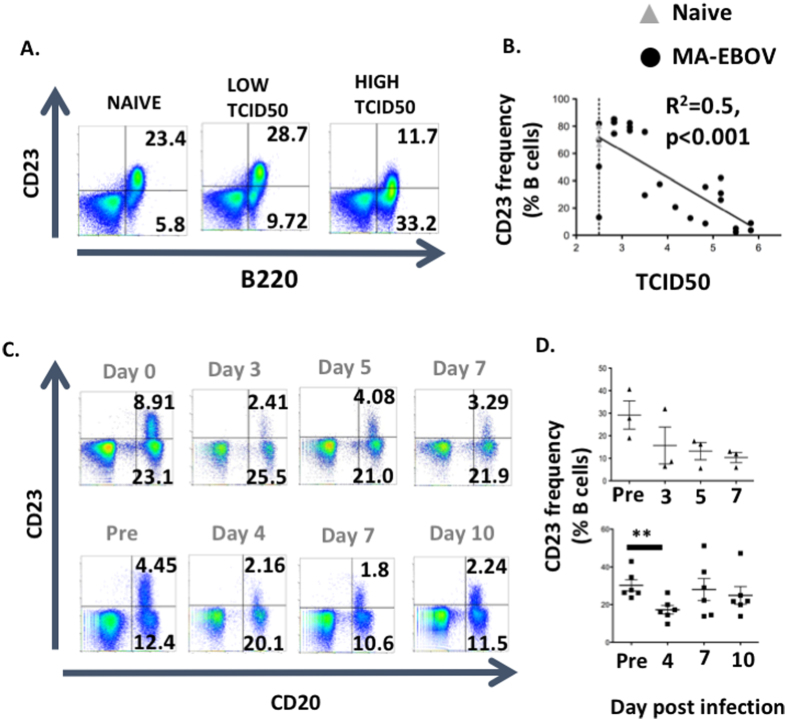
EBOV infection induces polyclonal B cell stimulation. CD23 surface expression on B cells of infected mice (**A,B**) and NHP (**C,D**) was analyzed by FACS. (**A,B**) CD23 expression was monitored on splenic B cells from surviving mice (n = 28), 7 days after MA-EBOV infection (1 LD50). Naïve uninfected mice (n = 4) were used as control. Representative plots (**A**) and correlations between CD23 and TCID50 are illustrated (**B**). Naïve mice are depicted by open triangle while MA-EBOV infected mice are represented by filled circles. Dotted lines indicated the limit of detection for TCID50 using the Spearman Kärber formula. Cumulative data from 4 separate experiments are illustrated (**C,D**) NHP were infected IM with ~1000LD50 EBOV. Monkey were bleed periodically and CD23 surface expression was measured at the indicated time-points. Representative plots (**C**) as well as summary data for CD23 surface expression (**D**) from untreated (top) and Zmapp treated (bottom) NHP are illustrated.

Next, polyclonal B cell activation by TLR ligands as well as release of sequestrated autoantigens which have both been linked to autoimmunity were investigated^18^,^19^. *In vitro,* antigen or TLR stimulation of B cells downregulated CD23 surface expression^20^. In order to demonstrate polyclonal B cells activation, CD23 surface expression was measured by FACS on the surface of B cells in both mice and NHP model of EBOV infection. Thirty-six mice were infected with 1LD50 MA-EBOV and 7 days post challenge, CD23 surface expression on splenic B cells were measured by FACS on surviving mice. Viremia inversely correlated with CD23 surface expression on splenic B cells (p < 0.001, R^2^ = 0.57) (Fig. 3A,B). To confirm these results, CD23 surface expression were also monitored on B cells from untreated and Zmapp treated NHP after ~1000 LD50 EBOV challenge. In both groups, challenge was associated with a marked decrease in CD23 level on circulating B cells (Fig. 3C,D). The extend of CD23 downregulation in both mouse and NHP model of infection, indicate that EBOV infection activates B cells independently of their antigen specificity, probably via TLR stimulation.

**Figure 4:**
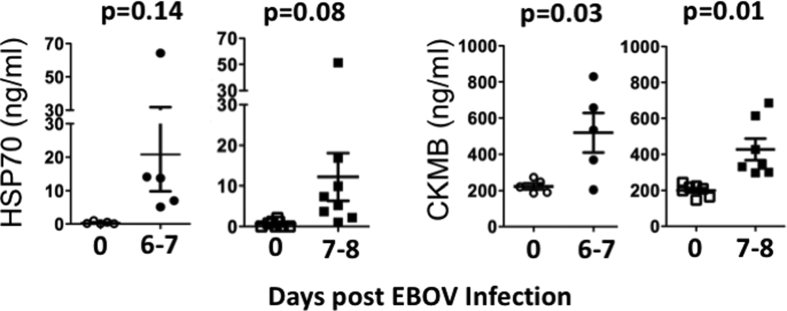
Autoantigens are secreted after EBOV challenge. Serum level of HSP70 and CK-MB were measured in control (left, n = 5) and ZMapp treated (right, n = 8) NHPs before (open symbols) and after (filled symbols) EBOV challenged. Samples were run in duplicate.

Finally, to investigate whether autoantigens secretion post-EBOV infection was implicated in the induction of autoantibodies, level of CK-MB and HSP70 were measured by ELISA in serum from EBOV challenged NHP (1000LD50). Both control and Zmapp treated NHP were studied. HSP70 was undetectable in sera from naive NHP. Conversely, 6–8 days post EBOV challenge, HSP70 serum level were detected in all control and half of Zmapp treated NHP, although the rise did not meet statistical significance between groups (p value 0.14 and 0.08 respectively) (Fig. 4). Conversely, CK was detectable in sera of naive NHP and serum levels of CK-MB significantly increase in both control and ZMAPP treated NHPs after infection (p value 0.03 and 0.01 respectively) (Fig. 4).

Taken together, the above results indicate that a combination of polyclonal B cells activation and release of sequestrated autoantigens may be responsible for EBOV induced autoimmunity.

### Antibodies accumulate in surviving mice’s tissues

**Figure 5:**
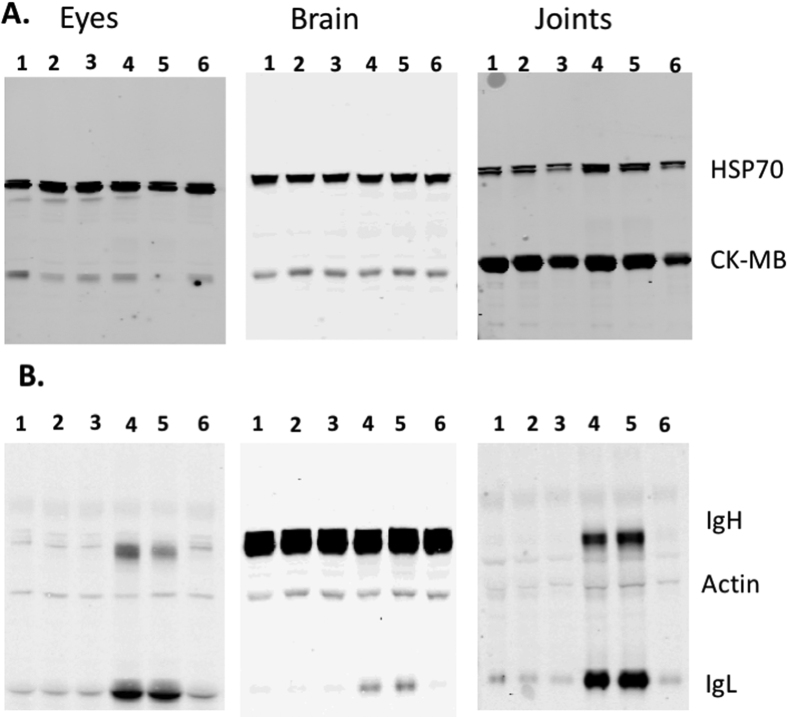
Antibodies accumulate in the eye, brain and joints of mice surviving MA-EBOV challenge. Level of HSP70 (70 kDa), CK-MB (43 kDa) (A.) as well as beta-actin (42 kDa), antibody light (25 kDa) and heavy chains (50 kDa) (B.) in tissues from naïve and MA-EBOV surviving mice were simultaneously assessed by WB. Beta Actin was used as loading control. For each blot, lane **1–3:** naïve mice; **4–6:** MA-EBOV surviving mice (3 weeks post challenge). Representative blots from 2 separate experiments.

For autoantibodies to be pathogenic, both autoantigen and autoantibodies have to be present at the site of damage^21^. Mental confusion (and sleeping disorders), ocular diseases as well as muscle and joint pains have frequently been reported in EBOV survivors^3^,^4^,^5^,^6^. To investigate the pathogenicity of autoantibodies in EBOV infection, antibodies accumulation and autoantigens level in the brain, eyes, and joints of mice surviving a MA-EBOV challenge was monitored. Eye, brain or joints lysates from naïve mice or mice 3 weeks post MA-EBOV challenge were analyzed by WB using a cocktail of mouse antibodies against beta actin and rabbit antibodies against HSP70 and CK-MB. Actin as well as Ig_H_ and Ig_L_ were detected using Dylight 800 conjugated goat anti mouse IgG, while HSP70 and CK-MB were detected using Dylight 650 conjugated anti rabbit IgG. No significant change in total HSP70 and CK-MB expression was detected in mice surviving MA-EBOV challenge (Fig. 5A). In contrast, there was a significant increase in antibody level in all tested tissues (eyes, brain, joints) from mice surviving MA-EBOV challenge compared with their naïve counterpart (Fig. 5B). Of note, a strong 50 kDa band, masking Ig_H_ levels, was observed in all brain samples. As a result, measurement of Ab accumulation in brain solely relied on Ig_L_ level (Fig. 5B).

These results suggest that antibodies accumulate in the eyes, brain and joints of mice which survived MA-EBOV challenge.

### Elevated autoantibodies levels in human EBOV survivors

The induction of autoimmunity by natural EBOV infection in humans was also investigated. The level of autoantibodies against HSPs, CK-MB and dsDNA were established and compared between healthy human donors, probable cases and RT-PCR confirmed EBOV cases. Antibodies level against EBOV GP was used as a positive control. For this study, sera from 16 healthy African donors were obtained from Kenya, where no EBOV outbreak has been reported. In addition, sera from 16 suspected or RT-PCR confirmed cases of EBOV infection, from the 2007, 2008/2009 outbreaks in Democratic Republic of Congo (DRC) and from the most recent outbreak in West Africa (2013/2016) were obtained (Table S2).

**Figure 6:**
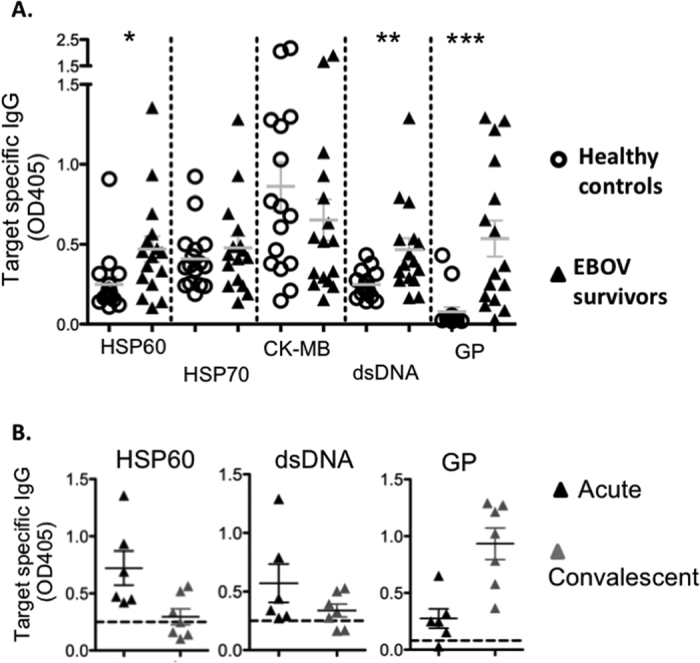
Level of autoantibodies against HSP60 and dsDNA are elevated in human EBOV survivors. (**A**) Humoral responses against autoantigens and EBOV GP were measured in healthy individuals [C] and in suspect or confirmed EBOV survivors [E] (n = 16/group). (**B**) Sera from 13 survivors were separated in the acute and convalescent groups. Samples collected within 32 days of symptoms onset were considered acute, while samples collected afterward were considered convalescent. Level of antibodies were analyzed by ELISA in 200 fold diluted sera while, anti EBOV GP antibody responses were measured in 2000 fold diluted sera. Samples were run in duplicate.

Antibodies against EBOV GP were detected in 2/16 (12.5%) and 11/16 (68.75%) of control and EBOV survivors respectively (Fig. 6A). It is worth noting that individual with EBOV GP antibodies were previously detected in population with no previous EBOV outbreak^22^,^23^. Interestingly, there was no statistical significant difference in antibody level against HSP70 or CK-MB between the two tested group (p values 0.44 and 0.3 respectively) (Fig. 6A). Conversely, EBOV survivors demonstrated a significant increase in autoantibodies against HSP60 and dsDNA (p values 0.02 and 0.006 respectively) (Fig. 6A). The above results strongly support the concept that natural EBOV infections also results in production of autoantibodies against HSP60 and dsDNA in humans. To investigate whether these autoantibodies persist in the sera of EBOV survivors, level of autoantibodies against HSP60 and dsDNA during the first month (32 days) post symptoms onset and during the convalescence phase were plotted. Antibody titers against EBOV GP were used as positive control. EBOV GP titers were higher during the convalescent compare with the acute phase, with high titers still detectable approximately one year post infection, which was the latest time point available (Fig. 6B). Conversely, level of autoantibodies against HSP60 and dsDNA were generally higher during the acute phase of infection and decline above background during the convalescent phase (Fig. 6B).

## Discussion

This study shows that elevated level of autoantibodies against HSP60 and dsDNA are present in mice, NHPs and humans surviving EBOV infection. This data presented also indicates that both polyclonal stimulation of B cell and secretion of autoantigens rather than antigen mimicry are involved in EBOV induced autoimmunity. It is worth pointing out that the strong correlation between HSP70 specific autoantibodies and GP specific antibodies in surviving NHP is probably due to the presence of host HSP70 in EBOV viral particles rather than antigen mimicry^24^. In human survivors, level of autoantibodies against HSP60 and dsDNA rose quickly after symptoms onset, then steadily decline during the convalescence phase. Initially, when levels of autoantibodies are rising, higher level of autoantibodies were associated with stronger humoral responses against EBOV GP. This correlation didn’t hold true in the later phase as autoantibodies titers decline, while humoral responses against EBOV GP keep on increasing.

It is worth noting that autoantibodies against additional unidentified autoantigens may exist in EBOV survivors. Furthermore, increased level of autoantibodies against HSP70 and CK-MB were detected in animal models of EBOV infection but not in human EBOV survivors. High background levels of these autoantibodies in human controls may in part be responsible for the observed lack of significant difference.

We hypothesized that the autoantibodies developed following EBOV infections are responsible for the PEVDS. Unfortunately, in the present study, PEDVS could not be correlated with autoantibody levels in animals or human survivors. Monitoring mental confusion, hearing loss, ocular diseases or even joint and muscle inflammation on live NHP following EBOV challenge would require specialized instruments not yet available in BSL-4 laboratories. Furthermore, tissues affected by PEVDS were not available from surviving NHPs preventing retrospective studies of PEVDS in NHPs. Finally, clinical information was unfortunately not available from the human survivors included in this study. Of note, PEVDS has been reported years post EBOV clearance from the circulation, and in this study, autoantibodies against dsDNA and HSP60 were only detectable for about 2 months post symptoms onset. The transient expression level of these autoantibodies in the blood may be due to antibody accumulation in various tissues. For autoantibodies to be pathogenic, both autoantigens and autoantibodies have to be present in the damaged tissues^21^. In the mouse model of EBOV infection, antibodies accumulate in the eyes, brain and joints of mice surviving MA-EBOV challenge, supporting a detrimental colocation and role of autoantibodies in ocular and cerebral diseases as well as joint pains observed in PEVDS. Unfortunately, antibody accumulation could not be assessed in NHP surviving EBOV challenge as biopsies from these tissues were not available. The eye, brain and inner ears are considered immune privilege sites. Antibodies accumulation in these tissues is indicative of damage to the blood-retina, blood-brain and blood-labyrinth barriers respectively^25^. Following entry, EBOV disseminates throughout the body into most tissues^26^ including the muscles, the eyes^4^,^7^ and the central nervous system (CNS)^8^. EBOV infection induces extensive cellular death^26^ which results in the release of cell-free DNA^27^. Furthermore, HSP60 is ubiquitously expressed in most tissues. Although HSPs are predominantly intracellular, they can translocate to the plasma membrane and even be secreted under various stress conditions including viral infections^13^,^14^,^28^. Therefore, EBOV replication is possibly responsible for the initial tissue damage that leads to HSP and cell free DNA secretion and ultimately loss of immune tolerance.

Clinical studies of EBOV survivors will be necessary to conclusive demonstrate the contribution of autoantibodies in PEVDS, as this cannot be demonstrated in animal models. It is worth noting that autoantibodies against HSP have previously been linked to various autoimmune diseases including ocular diseases^29^,^30^, rheumatoid arthritis^31^ and cerebral disorders^32^,^33^. More importantly, inducing tolerance against HSP70 or HSP60 prevents disease is multiple mouse models of autoimmunity^34^,^35^,^36^,^37^. Antibodies against dsDNA are also linked with autoimmune diseases. Detection of autoantibodies against dsDNA is required for diagnosis and severity classification of systemic lupus erythematosus (SLE)^15^.

In summary, the above study supports the concept that EBOV-induced autoimmunity is involved in PEVDS. This provides critical clues for treatment of PEVDS by pointing out the need for treatments either preventing autoimmunity induction or removing pathogenic autoantibodies generated during EBOV. Finally, the pathogenicity of autoantibodies against HSP60 and dsDNA in EBOV survivors will require further investigation in survivors.

## Methods

### Ethic statement

All infectious animal work was performed in the biosafety level 4 biocontainment laboratory at the Public Health Agency of Canada and approved by the Canadian Science Centre for Human and Animal Health Animal Care Committee following the guidelines of the Canadian Council on Animal Care. Animals were all acclimatized for at least 7 days prior to the start of any experiments. Animals were fed and monitored daily before and during the course of the experiments.

### Mice, NHP, viral challenge

Female 5–6 weeks old BALB/c mice were purchased from Charles River (Quebec, Canada). When indicated, mice were infected intraperitoneally (IP) with 200 ul containing 1LD50 (0.1 Foci Forming Unit) of MA-EBOV. This strain has been previously described^38^.

Surviving NHP sera were obtained from previous studies. The vaccinated NHP group consists of 5 cynomologus macaques immunized either intramuscularly (IM), orally or intranasally with 2 × 10^7^ PFU/ml recombinant VSVΔG/ZEBOV GP and challenged IM 28 days later with 1000 PFU EBOV Kikwit 1995^16^. Sera before vaccination, 21 days post VSVΔG/ZEBOV GP and 26 days post EBOV challenge were studied. Zmapp treated NHP have been previously described. Briefly, 12 rhesus macaques were challenged IM with 2512 PFU EBOV kikwit 1995 and treated with ZMapp starting 4 and 5 days post challenge^17^. Sera prior to and 28 days post viral challenge were analysed.

Sera from control animals from previous studies were used to measure CK-MB and HSP70 level post EBOV challenge as well as CD23 surface expression. One cynomologus macaques and 4 rhesus macaques were challenged IM with between 628 and 2512 PFU of EBOV kikwit 1995. Control animals received either PBS or control monoclonal antibodies post challenged^17^,^39^.

### Tissue lysis

Naïve mice were exsanguinated by cardiac bleed before euthanasia. Various tissues were then collected and lysed in Tris buffer containing 1% NP40 (Nocidet) (Sigma Aldrich, Oakville, Canada) and protease inhibitors cocktail (Roche diagnostic, Laval, Canada). Samples were lysed and homogenized using stainless steel bead and a Tissue Lyser (Qiagen, Toronto, Canada) for 10 mn at 30 Hz followed by an additional 30 mn under gentle agitation at 4 °C.

### Autoantibodies screen by ELISA

To monitor autoantibodies levels, 96 well plates were coated overnight at 4 °C with either 13 ug of lysate or 30 ng of purified proteins including Ebola virus (Zaire strain) GPΔTM, VP40 (IBT Biosciences, Gaithersburg, MD), HSP60 and HSP70 (Abcam, Toronto, Canada) as well as cardiac type creatine kinase MB (CK-MB) (Cedarlane, Burlington, Canada). Plates were blocked with PBS 5% milk prior to incubation with mice or NHP sera at dilutions indicated in the text. Alternatively, plates were incubated with 250 ng of the EBOV GP specific mAb which are part of the Zmapp therapeutic cocktail (13C6, 2G4 or 4G7)^17^. After two hour incubation 37 °C, plates were extensively washed and incubated for another hour with 25 ng of either HRP conjugated anti-mouse IgG or anti-human IgG antibodies (KPL, Gaithersburg, MD). After washing, plates were incubated with ABTS substrate (KPL). Absorbance was read at 405 nm using a plate reader (Molecular devices, Sunnyvale, CA). Each sample was run in triplicate.

For anti-dsDNA ELISA, 96 well plates were coated overnight at 4 °C with 300 ng of pCAGGS plasmid. Plates were blocked with 5% Bovine Albumin Fraction V (ThermoFisher scientific, Burlington, Canada) for 2 hrs. Diluted sera were incubated at room temperature for 1 hr. Plates were extensively wash, incubated with secondary antibodies and read as described above. Each sample was run in triplicate.

### Competition ELISA

For each well, diluted sera were pre-incubated in absence or presence of 10 times the amount of coating material of gamma irradiated EBOV or MARV virus for 1 hr at 37 °C. The serum/virus mixture was then added to well pre-coated with 30 ng of purified proteins or 300 ng plasmid. Serum binding to autoantigens was then analyzed as described above.

### Autoantibodies screening by WB

25 ug of tissue lysate was loaded per lane on a 4–12% Bis-Tris gel (ThermoFisher scientific, Burlington, Canada). Proteins were then transfer onto nitrocellulose membranes using the iblot system (ThermoFisher scientific). Membranes were blocked using TBS with 5% milk prior to overnight incubation with 20 ul of serum (1/500 dilution). After extensive washes, membranes were incubated with 1 ng of Dylight 800 conjugated goat anti-mouse IgG (Mandel scientific, Guelph, Canada), washed and analysed with an Odyssey Imager (Licor).

### HSP70, CK-MB and Antibodies level in tissues

Approximately 12 ug of eye and brain lysate and 20 ug of joint lysate were loaded per well of 4–12% Bis-Tris gels (ThermoFisher scientific). After separation, proteins were transferred onto nitrocellulose membranes using the iblot system (ThermoFisher scientific). Membranes were blocked using TBS with 5% milk prior to overnight incubation with 5 ng of mouse monoclonal anti-beta actin antibody, 11 ng rabbit anti mouse HSP70 and 10 ng rabbit anti mouse creatine kinase, all from Abcam. After extensive washes, membranes were incubated with 1 ng of Dylight 800 conjugated goat anti-mouse IgG and 1 ng Dylight 680 conjugated goat anti-rabbit IgG (Mandel scientific) and analyzed as described above.

### In gel digestion and Mass spectrometry

25 ug tissue lysate was loaded per lane on 4–12% gel (ThermoFisher scientific) and analysed by SDS-Page. Obtained gels were stained using Coomasie blue. Bands of the appropriate molecular weight were excised, destained, reduced, alkylated and trypsinised as previously described^40^. The resulting peptides were then analysed by nano-LC/MS/MS. Briefly The peptide fractions were separated on a C_18_-reversed phase column before analysis on a nano-flow Easy nLC I connected in-line to an LTQ Orbitrap XL mass spectrometer with a nanoelectrospray ion source. All spectra were processed using Proteome Discoverer (v2.0, Thermo Fisher Scientific) and database searching was done with Mascot v2.5 (Matrix Science).

### Flow cytometry

Anti-Human CD23 (M-L233), anti-mouse B220 (RA3-6B2) and CD23 (B3B4) antibodies were purchased from BD Biosciences (San Jose, CA) while anti-human CD20 (2H7) antibodies were from Biolegend (San Jose, CA). LIVE/DEAD fixable far red Dead cell stain kit (Life technologies, Burlington, Canada) was used to differentiate live from dead cells.

A single cell suspension was obtained from each individual mice then lysed using ACK lysis buffer (Life technologies) according to the manufacturer’s instructions. Splenocytes were then stained using a viability dye and a cocktail of CD23 and B220 antibodies.

For NHP samples, 100 ul of blood was stained with a viability dye and a cocktail of CD23 and CD20 antibodies. Red blood cells were then lysed using ACK lysis buffer before samples fixation using Cytofix/cytoperm (BD Biosciences).

Samples were run onto an LSRII (BD Biosciences, San Jose, CA) and analyzed using Flow Jo (version 10, Treestar).

### TCID50

Serially diluted blood samples were added on 90–95% confluent VeroE6 cells for an hour at 37 °C. Blood was then replaced with DMEM supplemented with 2% FBS and 1X Penicillin/streptomycin (Life technologies, Burlington, Canada). After 2 weeks incubation, each well was scored for the presence or absence of CPE. The Spearman Kärber formula was then used to calculate TCID_50_/mL. Of note the limit of detection of our assay is log_10_ 2.5 TCID_50_/mL.

### HSP70 and CK protein level in NHP sera

HSP70 and CK ELISA kit were purchased from Abcam. Serum levels were measured according to the manufacturer’s instructions.

### Human samples

Sera from 16 healthy, HIV and HCV negative donors were obtained from Kenya, following approval from ethic boards from the University of Manitoba and Kenyatta National Hospital REB in Nairobi. All donors provided individual inform consent. Sera from probable cases of EBOV infection were obtained from the 2007, 2008–2009 EBOV outbreak in DRC, as well as RT-PCR confirmed cases from the 2013–2015 outbreak in West Africa. Individuals were categorized as probable cases based on WHO guidelines. Informed consent was not obtained from these individual patients. Ethics approval was obtained from local Governments (DR Congo and Sierra Leone) Ethic Review Board to use, analyze, and publish controlled, unidentified, anonymous data collected during diagnostic testing. All experiments described in this manuscript were carried out in accordance with relevant guidelines and regulations.

### Statistical analysis

Linear regression analysis was performed for correlation between autoantibodies levels and the other tested parameters. Increase in antibody levels were analyzed by 1 way ANOVA follow by the Dunnett or Bonferroni test in VSV vaccinated and Zmapp treated NHP respectively. Difference in HSP70 and CK-MB serum levels were analyzed using paired t test while for each target difference in antibody levels between healthy donors and EBOV survivors were analyzed using unpaired t test. All statistical analyses were performed using GraphPad Prism version 5.03 software. Throughout the manuscript *, ** and *** indicates statistically significant differences with p < 0.05, 0.01 and 0.001 respectively.

## Additional Information

**How to cite this article:** Fausther-Bovendo, H. *et al*. Ebola virus infection induces autoimmunity against dsDNA and HSP60. *Sci. Rep.* **7**, 42147; doi: 10.1038/srep42147 (2017).

**Publisher's note:** Springer Nature remains neutral with regard to jurisdictional claims in published maps and institutional affiliations.

## Supplementary Material

Supplementary Figures
