# Supplementary material for: Ebola virus infection induces autoimmunity against dsDNA and HSP60
Source: Sci Rep. 2017 Feb 9;7:42147. doi: 10.1038/srep42147 (PMC5299614; doi:10.1038/srep42147)
Supplement: Supplementary Figures [file srep42147-s1.pdf]

Ebola virus infection induces autoimmunity against dsDNA and HSP60

H. Fausther-Bovendo[1,2], X. Qiu[1,2], S McCorrister[3], G. Westmacott[3], P. Sandstrom[3,4], C. Castilletti[5], A. Di Caro[5], G. Ippolito [5], G. P. Kobinger*[2, 6, 7]

Supplemental Information

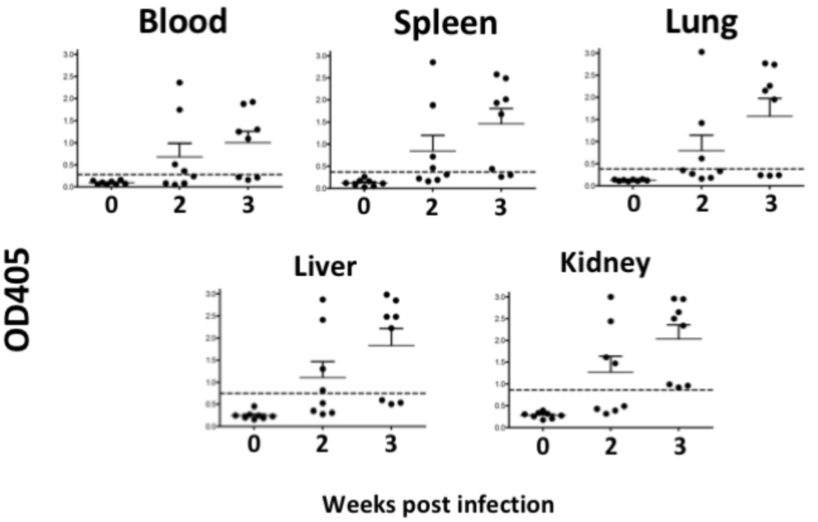

Figure S1: Autoantibodies are detected in some MA-EBOV survivors

50 fold diluted sera from naïve mice (**0**) and MA-EBOV infected mice 2 or 3 weeks after challenge were screened from the presence of autoantibodies against various tissues by ELISA. Cumulative results from 2 separate experiments are depicted (n=8 per group total). Each sample was run in triplicate.

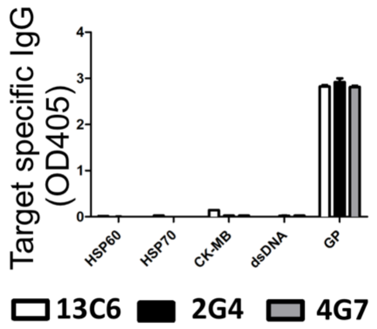

Figure S2: The mAb within Zmapp (13C6, 2G4, 4G7) do not cross-react with the tested autoantigens

Binding of the EBOV GP specific mAb 13C6, 2G4 and 4G7 to various autoantigens was tested by ELISA. EBOV GP was used as positive control. Each condition was run in triplicate.

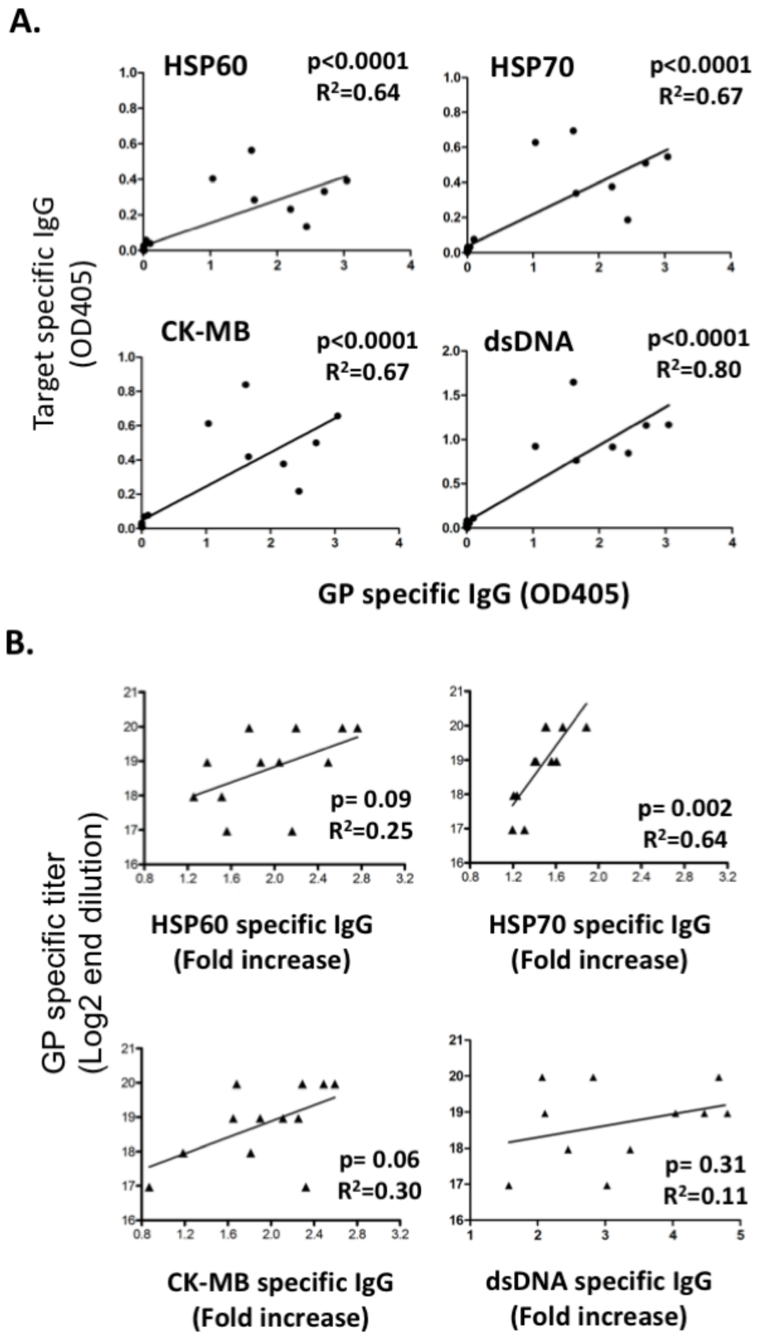

Figure S3: Correlation between autoantibody level and GP specific antibodies.

Correlation between EBOV GP and autoantibody titer were analysed in surviving mice (**A.**) and Zmapp treated NHP (**B.**) following MA-EBOV and EBOV challenge. (**A.**) Correlation between humoral response against EBOV GP (2000x dilution) and autoantibodies level in MA-EBOV

survivors are illustrated (n=16). (**B.**) Correlation between GP specific antibody titer and autoantibodies fold increase are represented. Representative data from 2 separate experiments.

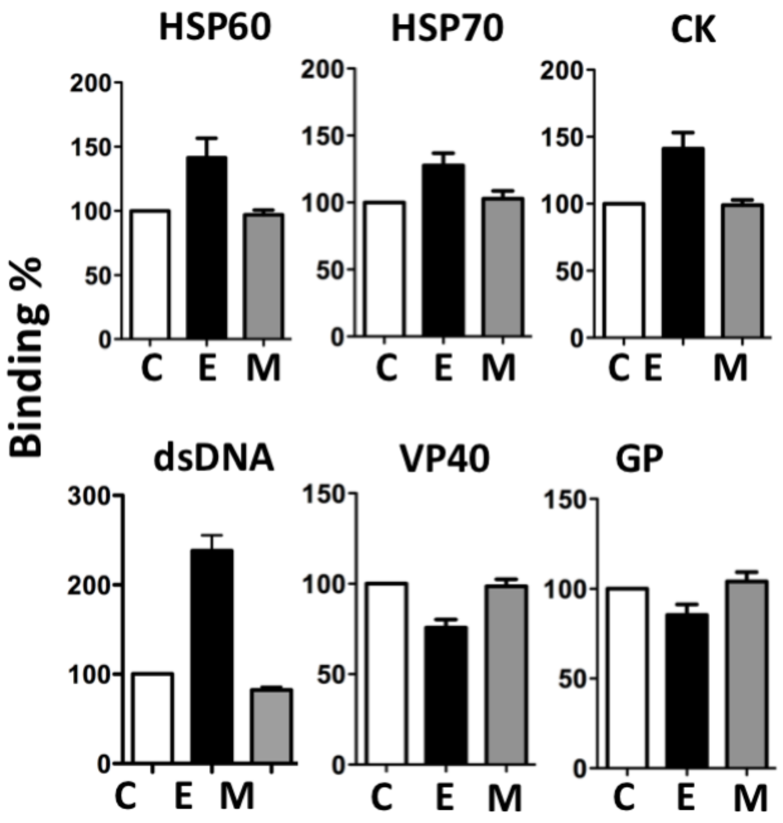

Figure S4: Autoantibody are not caused by antigen mimicry.

Binding to autoantigens as well as EBOV VP40 and GP was monitored in presence or absence of 10-fold gamma irradiated EBOV or MARV viral particles using various dilutions of sera from MA-EBOV surviving mice. Competition ELISA were performed using 200 fold diluted sera for autoantigen as well as 800 and 4000 fold diluted sera for VP40 and GP respectively. Each sample (n=7 per group) was run in triplicate. C = control, E= EBOV, M=MARV.

		Protein Name	accession number	molecular weight (kDa)	Quantitative values- Normalize total Spectra			
					Joint	Muscle	Ear	Brain
Target A (~60 kDa)	1	Serum Albumin	ALBU_MOUSE	69	163	181	3330	N.A
	2	Sarcoplamic/endoplasmic reticulum calcium ATPase 1	AT2A1_MOUSE	109	50	95	35	N.A
	3	**60 kDa heat shock protein**	**CH60_MOUSE**	**61**	**54**	**20**	**53**	**N.A**
	4	Stress induced phosphoprotein-1	STIP-1	63	30	46	34	N.A
	5	Phosphoglucomutase	PGM1_MOUSE	61	41	32	28	N.A
	6	Leukotriene A-4 hydrolase	LKHA4_MOUSE	69	37	25	30	N.A
	7	very long chain specific acyl-CoA dehydrogenase	ACADV_MOUSE	71	29	41	21	N.A
	8	Carnitine 0-acetyltransferase	CACP_MOUSE	71	26	44	22	N.A
	9	Pyruvate kinase PKM	KPYM_MOUSE	58	29	39	24	N.A
	10	Dihydrolipoyllysine-residue acetyltransferase component of pyruvate dehydrogenase complex	ODP2_MOUSE	68	24	34	29	N.A
Target B (~40 kDa)	1	**Creatine kinase M-type**	**KCRM_MOUSE**	**43**	**235**	**440**	**296**	**14**
	2	**Creatine kinase B-type**	**KCRB_MOUSE**	**43**	**23**	**11**	**46**	**98**
	3	Phosphoglycerate kinase 1	PGK1_MOUSE	45	50	70	42	7

	4	Succinyl-CoA ligase subunit beta	SUCB1_MOUSE	50	43	52	41	22
	5	Creatine kinase S type	KCRS_MOUSE	47	65	68	52	3
	6	Aspartate aminotransferase	AATC_MOUSE	46	25	34	30	31
	7	Fructose-biphosphate aldolase A	ALDOA_MOUSE	39	24	48	24	40
	8	Long chain specific acyl-CoA dehydrogenase	ACADL_MOUSE	48	27	29	22	13
	9	Acetyl-CoA acetyltransferase	THIL_MOUSE	45	20	22	20	27
	10	Aspartate aminotransferase	AATM_MOUSE	47	13	11	23	36

Table S1: Protein identification by LC-MS/MS

Proteins were identified using lysate from joint, muscle, brain as well as inner ear and temporal bone. The 10 most abundant proteins, as well as their relative level in the various lysate are illustrated. For each target, the proteins with the appropriate molecular weight and expression pattern similar to the one observed by western blot are highlighted in bold.

Sample number	Sample Origin	Symptoms onset (yyyy-mm-dd)	Sampling date (yyyy-mm-dd)	Time from onset (days)	GP (0D405)	HSP60 (0D405)	dsDNA (0D405)
DRCM 07 011	2007, DRC	Unknown	Unknown	Unknown	0.09	0.37	0.54
DRCM 07 035	2007, DRC	Unknown	Unknown	Unknown	0.12	0.29	0.37
09 FVH 037	2008-2009, DRC	2008-12-03	2009-01-24	52	1.29	0.33	0.39
09 FVH 038	2008-2009, DRC	2008-12-03	2009-01-24	52	0.79	0.25	0.53
09 FVH 039	2008-2009, DRC	2008-12-23	2009-01-24	32	0.32	0.47	0.44
09 FVH 040	2008-2009, DRC	2008-12-03	2009-01-24	52	0.58	0.57	0.50
09 FVH 041	2008-2009, DRC	2009-01-15	2009-01-22	7	0.15	0.45	0.34
09 FVH 044	2008-2009, DRC	2009-01-22	2009-01-26	4	0.03	0.42	0.29
09 FVH 050	2008-2009, DRC	2008-12-25	2009-01-02	8	0.25	0.69	0.79
09 FVH 054	2008-2009, DRC	2008-12-24	2009-01-03	10	0.65	1.36	1.29
09 FVH 060	2008-2009, DRC	Unknown	2009-01-05	Unknown	0.18	0.45	0.76
09 FVH 063	2008-2009, DRC	2009-01-18	2009-02-06	19	0.25	0.9345	0.2745
Serum 6001	2013-2016, West Africa	2015-02-10	2016-01-13	337	1.27	0.14	0.17
Serum 6050	2013-2016, West Africa	2015-05-13	2015-08-27	106	0.37	0.16	0.28
Serum 6057	2013-2016, West Africa	2014-12-16	2015-11-19	338	1.22	0.10	0.17
Serum 906	2013-2016, West Africa	2014-11-24	2015-02--05	73	1.02	0.52	0.33

Table S2: Characteristic of samples from confirm or suspected EBOV survivors.

Serum samples from suspected EBOV survivors were obtained from 3 separate outbreaks including the most recent one in West Africa. The origin, symptoms onset and sampling date as well as antibody level against EBOV GP, HSP60 and dsDNA are indicated for each sample.
